# Continuing Contributions of Field Epidemiology Training Programs to Global COVID-19 Response

**DOI:** 10.3201/eid2813.220990

**Published:** 2022-12

**Authors:** Elizabeth Bell, Camille Mittendorf, Erika Meyer, Olivia Barnum, Carl Reddy, Seymour Williams, Henry Baggett, Reina Turcios-Ruiz

**Affiliations:** Centers for Disease Control and Prevention, Atlanta, Georgia, USA (E. Bell, C. Mittendorf, E. Meyer, O. Barnum, S. Williams, H. Baggett, R. Turcios-Ruiz);; Task Force for Global Health, Decatur, Georgia, USA (C. Reddy);; Training Programs in Epidemiology and Public Health Interventions Network (TEPHINET), Decatur (C. Reddy)

**Keywords:** COVID-19, FETP, Field Epidemiology Training Programs, global health security, workforce development, field epidemiology, COVID-19 response, preparedness, workforce capacity building, WHO Response Pillars, WHO COVID-19 Response Strategy, International Health Regulations, pandemic, pandemic response, coronavirus, SARS-CoV-2

## Abstract

We documented the contributions of Field Epidemiology Training Program (FETP) trainees and graduates to global COVID-19 preparedness and response efforts. During February–July 2021, we conducted surveys designed in accordance with the World Health Organization’s COVID-19 Strategic Preparedness and Response Plan. We quantified trainee and graduate engagement in responses and identified themes through qualitative analysis of activity descriptions. Thirty-two programs with 2,300 trainees and 7,372 graduates reported near-universal engagement across response activities, particularly those aligned with the FETP curriculum. Graduates were more frequently engaged than were trainees in pandemic response activities. Common themes in the activity descriptions were epidemiology and surveillance, leading risk communication, monitoring and assessment, managing logistics and operations, training and capacity building, and developing guidelines and protocols. We describe continued FETP contributions to the response. Findings indicate the wide-ranging utility of FETPs to strengthen countries’ emergency response capacity, furthering global health security.

Field Epidemiology Training Programs (FETPs), modeled on the Epidemic Intelligence Service (EIS) of the US Centers for Disease Control and Prevention (CDC), are competency-based training programs designed to strengthen national and regional health security infrastructure and enhance the epidemiologic capacity of the public health workforce (*1*–*3*). FETP expands on the EIS model with 3 tiers of training of increasing duration and complexity: 3–4 months of frontline, 5–9 months intermediate, and 2 years of advanced training (*1*,*4*,*5*). The Global Health Security Agenda (GHSA) was launched in 2014 to strengthen countries’ capacities for detection, response, and prevention of public health threats and to accelerate progress toward meeting the World Health Organization (WHO) International Health Regulations 2005 (IHR 2005) targets (*6**,**7*).

The COVID-19 pandemic highlighted global vulnerability to infectious-disease threats. The widespread and sustained response it required further emphasized the need for strengthened field epidemiology workforce capacity across all regions and levels of public health systems. Although recent reports feature FETPs’ response to COVID-19 (*8*–*10*), a need for global-level documentation remains. We sought to document and characterize the contributions of FETP trainees and graduates to COVID-19 preparedness and response around the globe at 13 months into the global pandemic.

## Study Design and Methods

We conducted and presented findings from our first survey of program directors of FETPs around the world in March–April 2020 (*11*); we conducted a second survey of program directors during February–April 2021. Those surveys included questions about which tiers of FETPs were implemented and about the engagement of program trainees and graduates in COVID-19 response activities categorized according to the COVID-19 Preparedness and Response Plan’s 10 strategic pillars (*12*) (Table 1). The pillars are the following: pillar 1, country-level coordination, planning, and monitoring; pillar 2, risk communication and community engagement; pillar 3, surveillance, rapid response teams, and case investigation; pillar 4, point of entry; pillar 5, national laboratories; pillar 6, infection prevention and control; pillar 7, case management; pillar 8, operational support; pillar 9, maintaining essential health services and systems; and pillar 10, vaccination (against COVID-19). Pillars 9 and 10 were added to the original 8 (*13*). We asked each program director for the total number of graduates and current trainees in their program. We asked if persons in any stage of their FETP training (trainees) or those who successfully completed their graduation requirements (graduates) or both were engaged in response activities and asked for brief descriptions of those activities.

**Table 1:** Ten pillars of the World Health Organization Strategic Preparedness and Response Plan for COVID-19*

Pillar no.	Public Health Preparedness and Response area
1	Coordination, planning, financing, and monitoring
2	Risk communication, community engagement and infodemic management
3	Surveillance, epidemiologic investigations, contact tracing, and adjustment of public health and social measures
4	Points of entry, international travel and transport, and mass gatherings
5	Laboratories and diagnostics
6	Infection prevention and control, and protection of the health workforce
7	Case management, clinical operations, and therapeutics
8	Operational support and logistics, and supply chains
9	Maintaining essential health services and systems
10	Vaccination

*As of February 2021. Source: World Health Organization (*11*).

We distributed invitations to respond to the online SurveyMonkey (Momentive Inc., https://www.surveymonkey.com) survey to 92 FETP program directors via email in February 2021 in coordination with the Training Programs in Field Epidemiology and Public Health Interventions Network (TEPHINET), a global network of FETPs. If a program director had responded to our first survey in 2020, they were asked to report on the activities conducted since that submission. If a program director had not responded to the first survey, we asked them to report on all the activities in which FETP trainees or graduates had engaged for COVID-19 preparedness or response. We followed up on incomplete or duplicate responses by email or telephone calls with respondents during April–July 2021 to complete or reconcile responses.

### Quantitative Analysis

We mapped the responding programs to describe the geographic distribution. We analyzed selected characteristics of responding programs: years between the establishment of the program and July 2021, and days between the report of the first case of COVID-19 in the country and the date of survey response. We calculated medians and reported minimum and maximum values aggregated by WHO region. We tabulated responses and calculated by WHO region and WHO pillar percentages of programs reporting FETP trainee or graduate engagement in COVID-19 preparedness or response activities by using Microsoft Excel (Microsoft, https://www.microsoft.com).

### Qualitative Analysis

Four team members conducted content analysis on qualitative responses using MaxQDA (VERBI Software, https://www.maxqda.com). Each analyst reviewed the original codebook used for the qualitative analysis of the responses to our first survey (*11*). After reviewing all responses, we updated the codebook to reflect novel responses, new codes, new themes, and the activities corresponding to the 2 new response pillars. The 4 staff met weekly to reach consensus on new codes, consolidate codes, and identify themes across the 10 WHO pillars with appropriately illustrative quotes. Some survey respondents answered in their primary language; bilingual CDC staff translated responses in French, Portuguese, and Spanish, and we used Google Translate (https://translate.google.com) for responses in Ukrainian and Chinese.

## Results

### Quantitative Findings

**Figure 1:**
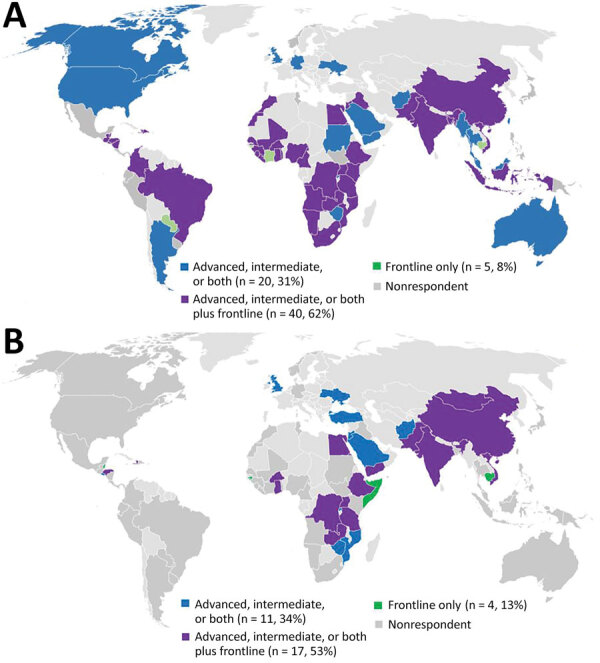
Geographic distribution of Field Epidemiology Training Programs invited to respond to a survey about their contributions to global COVID-19 response. Responding programs are identified by the tiers of training implemented. A) Programs invited to respond to the 2021 survey (n = 92). B) Programs invited to the 2020 survey (n = 88; Hu et al. [*10*]).

Of 92 program directors invited to the survey, 32 (35%) responded, reporting on COVID-19 preparedness and response activities in 69 countries across all WHO regions (Figure 1, panel A). Thirty of the respondents represented national programs and 2 represented regional programs, 1 serving 24 countries in the Caribbean (Americas region, Pan American Health Organization [PAHO]), and the other 19 countries covered by the WHO Regional Office for the Eastern Mediterranean (EMRO). Four programs in Belize, Haiti, Egypt, and Ukraine implemented training nationally but were also served by a regional program. Of the 32 responding programs, 17 (53%) were implementing frontline training as well as advanced, intermediate, or both tiers; 6 programs were implementing all 3 tiers of field epidemiology training. Among responding programs, 4 (13%) were implementing frontline only.

Half of the programs that responded to this survey were >10 years old, and nearly all were in countries in which the earliest known COVID-19 case was >1 year prior (Table 2). Only the 4 reporting programs in the PAHO region had yet to surpass the 1-year mark between the earliest reported case of COVID-19 and responding to this survey. Programs <5 years old from 3 WHO regional offices responded; those countries were Burkina Faso (Regional Office for Africa [AFRO]), Ukraine (Regional Office for Europe [EURO]), and Afghanistan and Somalia (EMRO). Of note, the Somalia FETP established frontline training in 2021, during the COVID-19 pandemic. The 32 programs reported a combined total of 2,300 trainees and 7,372 graduates.

**Table 2:** Selected characteristics of the Field Epidemiology Training Programs that responded to surveys about COVID-19 response, 2020–2021*

WHO regional office	This study				Survey 1†		
	No. reporting country programs (no. invited)	Median age of program, y (range)	Median days since first reported COVID-19 case in country (range)‡		No. reporting country programs (no. invited)	Median age of program, y (range)	Median days since first reported COVID-19 case in country (range)‡
Africa	11 (30)	11 (3–28)	405 (322–491)		24 (27)	8 (2–27)	19 (3–35)
Eastern Mediterranean	7 (12)	15 (0–32)	414 (376–508)		9 (11)	10 (1–31)	33 (14–51)
Europe	4 (9)	9 (3–10)	446 (411–498)		6 (9)	11 (2–25)	47 (23–52)
Americas	5 (21)	20 (10–20)	340 (330–399)		15 (22)	19 (3–69)	27 (11–74)
Southeast Asia	1 (6)	20 (20–20)	448 (448–448)		5 (7)	19 (2–40)	34 (16–74)
Western Pacific	4 (14)	11 (10–20)	425 (407–565)		6 (12)	18 (9–36)	73 (56–105)
All programs	32 (92)	11 (0–32)	412 (322–565)		65 (88)	11 (1- 69)	25 (3–105)

*One regional program in Europe and 1 in the Americas were excluded from calculation of days to survey response since first COVID-19 case was reported. Refer to Table 1 for numbers in each region by survey. †Survey 1, Hu et al. (*10*). ‡Regional programs serving multiple countries and four programs (Guinea-Bissau, Mozambique, Sierra Leone, and Yemen) that responded to the survey before the first COVID-19 case was reported in their country were not included in the calculation of days to survey response since first COVID-19 case was reported

**Figure 2:**
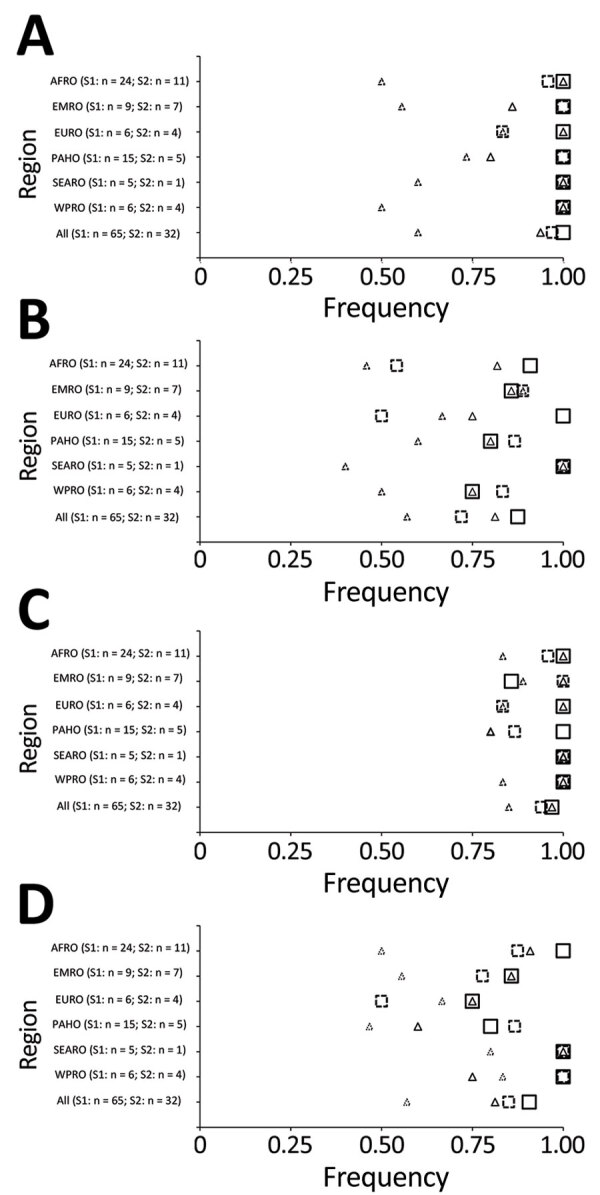
Field Epidemiology Training Programs (FETPs) reporting trainee or graduate support to COVID-19 preparedness and response by WHO response pillar and WHO regional office (AFRO, Africa; EMRO, Eastern Mediterranean; EURO, Europe; PAHO, Americas; SEARO, Southeast Asia; WPRO, Western Pacific). Programs indicating engagement of FETP trainees, graduates, or any FETP involvement (trainees or graduates) are shown. A) Pillar 1, country-level coordination. B) Pillar 2, risk communication and community engagement. C) Pillar 3, surveillance, response teams, case investigations. D) Pillar 4, points of entry. S1, survey 1; S2, survey 2; WHO, World Health Organization.

**Figure 3:**
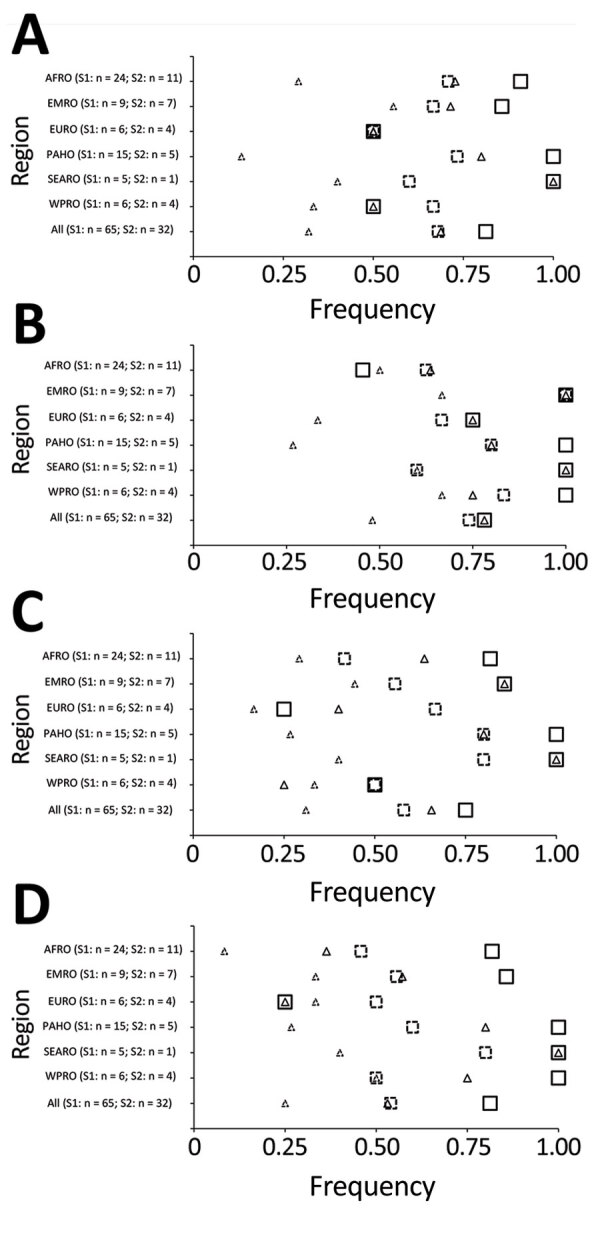
Field Epidemiology Training Programs (FETPs) reporting trainee or graduate support to COVID-19 preparedness and response by WHO response pillars 5, 6, 7 and 8, and by WHO regional office (AFRO, Africa; EMRO, Eastern Mediterranean; EURO, Europe; PAHO, Americas; SEARO, Southeast Asia; WPRO, Western Pacific). Programs indicating engagement of FETP trainees, graduates, or any FETP involvement (trainees or graduates) are shown. A) Pillar 5, national laboratories. B) Pillar 6: infection prevention and control. C) Pillar 7, case management. D) Pillar 8, operational support and logistics. S1, survey 1; S2, survey 2; WHO, World Health Organization.

**Figure 4:**
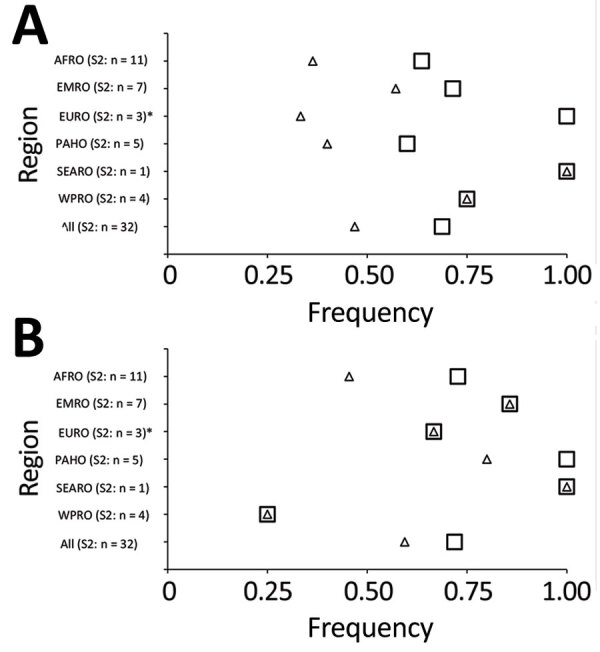
FETPs reporting trainee or graduate support to COVID-19 preparedness and response by WHO response pillars 9 and 10, and by WHO regional office (AFRO, Africa; EMRO, Eastern Mediterranean; EURO, Europe; PAHO, Americas; SEARO, Southeast Asia; WPRO, Western Pacific). Programs indicating engagement of FETP trainees, graduates, or any FETP involvement (trainees or graduates) are shown. A) Pillar 9, maintaining essential health services and systems. B) Pillar 10, vaccine country readiness and delivery. S1, survey 1; S2, survey 2; WHO, World Health Organization.

All 32 responding programs reported engagement of FETP trainees and graduates in all pillars of WHO response activities. The most frequently reported pillars of engagement for trainees or graduates, in order of decreasing frequency, were WHO pillar 3, surveillance, rapid response teams, and case investigation; pillar 1, coordination, planning, financing, and monitoring; pillar 2, risk communication and community engagement; and pillar 4, points of entry (Figure 2). Engagement of FETP trainees or graduates variable in activities corresponding to pillar 5, national laboratories; pillar 7, case management; pillar 6, infection prevention and control; and pillar 8, operational support (Figure 3). More programs reported engagement of graduates than reported engagement of trainees in response activities. Most evident of this trend were reports of engagement in activities of pillar 8, operational support and logistics; pillar 7, case management; and pillar 9, maintaining essential health services and systems. Notable exceptions to the more frequent engagement of graduates than trainees were in the EMRO region, where programs reported more trainees than graduates engaged in pillar 3, surveillance, response teams and case investigations; in the AFRO region in pillar 6, infection prevention and control activities; and in the EURO region in pillar 7, case management. Although pillar 9, maintaining essential health services and systems, and pillar 10, vaccination, were introduced in the updated WHO response plan of February 2021, >25% of programs reported that trainees and graduates were involved in activities of these new pillars (Figure 4).

### Qualitative Findings

Six themes emerged during content analysis that illustrate the contributions of FETPs to COVID-19 preparedness and response a year into the pandemic (Table 3). We identified these themes from the activity descriptions across multiple WHO pillars.

**Table 3:** Main themes identified from the descriptions of 10 pillars of World Health Organization response activities provided by Field Epidemiology Training Programs

Theme	Pillar
Epidemiology and surveillance activities	1, 2, 3, 4, 6, 7
Leading risk communication efforts	1, 2, 4, 7, 10
Monitoring and assessment activities	4, 5, 6, 8, 9, 10
Managing logistics and operations	1, 5, 6, 7, 8, 9, 10
Training and capacity building	1, 2, 3, 4, 5, 6, 7, 8, 10
Developing guidelines and protocols	1, 4, 5, 6, 7, 9, 10

#### Theme I: Epidemiology and Surveillance

Respondents commonly described epidemiologic and surveillance activities. This quote from Ethiopia captures the myriad ways FETPs are used: “Residents [i.e., trainees] are involved in case investigation […] and outbreak investigation, school reopening preparedness assessment. The graduates report surveillance data to the next level and analyze and report trends of diseases. They provide orientations to surveillance focal persons on the reporting mechanism, case definitions, reporting formats, and investigation procedures. Residents and graduates have supported serosurveillance and [severe acute respiratory infections] sentinel site surveillance at hospitals.” Several programs across the regions also reported that their trainees or graduates assisted in the development of the standard case definition for COVID-19 and led healthcare-associated infection investigations.

#### Theme II: Leading Risk Communication

When reporting on trainee and graduate risk communication activities, commonly reported work was medium-specific (staffing call centers, providing press interviews, posting on social media, etc.) or target population-specific messaging (healthcare workers, travelers, administrative officials, etc.). In Rwanda, “Advanced graduates provided radio and television interviews to disseminate public health messages.” In Egypt, trainees “[developed] timely and transparent communication messaging and materials for public regarding COVID-19 enquiries” and graduates “[developed] and updated the risk communication strategy, […detected] and quickly respond to misinformation and rumors.” Graduates were more commonly involved in the development of strategic planning or liaising with government officials—especially those who are employed at the ministry of health—whereas trainees were more frequently reported to be involved in direct interfacing with the public through public hotlines and social media. Graduates in Burkina Faso conducted “COVID media training [and] sensitization of leaders (community, religious and political) on COVID.”

#### Theme III: Monitoring and Assessment Activities

FETPs supported infection prevention and control activities for public and private institutions such as schools and companies (in Tanzania and Rwanda), risk assessments for healthcare facilities and schools (in India, Ethiopia, and Zimbabwe), and “monitoring and audit of infection prevention and control practices and feedback at hospital level” (in Egypt).

Graduates in El Salvador worked on event-based monitoring. Both graduates and trainees in Turkey and Ukraine monitored case numbers, surveillance data, and laboratory testing data to evaluate surveillance methods. In India, the COVID-19 vaccine rollout also provided opportunities for graduates to conduct “[monitoring] and supervision [of] vaccine rollout in states” and do “field monitoring of surge staff.”

#### Theme IV: Managing Logistics and Operations

FETP trainees and graduates managed logistics and operations at all levels, from testing and sampling to vaccine supply chain management, liaising between different institutions, and organizing staff deployments. In Zimbabwe, graduates worked on “adopting and disseminating SOPs [Standard Operating Procedures]… for specimen collection, management, and transportation for COVID-19 diagnostic testing.”

#### Theme V: Training and Capacity Building

FETP trainees and graduates were heavily involved in efforts to train and build COVID-19–related response capacity across sectors and levels of society. The data showed that from the community level (such as in Uganda, where graduates conducted “training of village health teams of community-based health surveillance”) all the way to the national and state levels (as in India, where trainees and graduates conducted “cascade training of national and state level officials on IPC [Infection Prevention and Control]”), their expertise was widely required. Programs reported their participation in training for the following response-related activities: point-of-entry screening, infection prevention and control at healthcare facilities and in the community, case management, specimen collection, and the incident management system.

FETP trainees and graduates served as trainers for vaccine-related rollout activities. They contributed to training on cold-chain standards (Rwanda), training healthcare workers on how to administer the vaccine (Jordan); and “training on abnormal response monitoring,” also known as adverse events monitoring (China).

#### Theme VI: Developing Guidelines and Protocols

FETP trainees and graduates were engaged in developing guidelines and protocols. They developed standard operating procedures and participated in national-level strategic planning, particularly for the preservation of essential health services and vaccine rollout. Their wide participation in vaccine-related planning was illustrative, as in this example from China: “FETP participants were integrated into the National Immunization Centre Vaccine Task Force to participate in the Vaccination Information Group.” Drafting case-management guidelines were also reported by many programs, such as in Jordan where both graduates and trainees “[established] guidelines to deal with suspected cases coming to Jordan and confirmed as well” and “were responsible for updating the management guidelines as soon as it needed and follow up [on] admitted cases.”

## Discussion

We documented the diverse contributions of FETP trainees and graduates to COVID-19 preparedness and response activities 1 year into the pandemic, across all WHO regions and response pillars, including the new pillar 9: maintaining essential health services and systems, and pillar 10: vaccination. Programs more commonly reported graduate than trainee engagement. Through content analysis, common themes emerged describing active engagement and vital roles in all types of activities of COVID-19 preparedness and response. The more frequent reporting of trainees and graduates working in specific pillars and the emerging themes reflect the core competencies of the advanced and intermediate tiers of FETPs (Table 4). The FETPs’ core competencies of epidemiologic methods, communication, and management and leadership were closely aligned with the pillars of most frequently reported trainee and graduate engagement: pillar 3, surveillance, rapid response teams, and case investigation; pillar 1, coordination, planning, financing, and monitoring; pillar 2, risk communication and community engagement; and pillar 4, points of entry. FETP trainees and graduates were also reported as involved in activities of the 2 new pillars in the revised WHO response plan (strengthening essential health services, and vaccination activities). FETPs’ contributions to these 2 pillars demonstrated that trainees and graduates can leverage their skills and knowledge to take on related response activities, likely with additional orientation as needed.

**Table 4:** Competencies in Field Epidemiology Training Programs by public health topic area*

Competency	Activity
Epidemiologic methods	Use epidemiologic practices to conduct studies that improve public health program delivery; respond to outbreaks
Biostatistics	Analyze epidemiologic data using appropriate statistical methods
Public health surveillance	Set up, manage, and evaluate a public health surveillance system
Laboratory and biosafety	Use laboratory resources to support epidemiologic activities
Communication	Develop written public health communications; develop and deliver oral public health communications
Computer technology	Use computers for specific applications relevant to public health practices
Management and leadership	Manage a field project; manage staff and resources; be an effective team leader and member; manage personal responsibilities
Prevention effectiveness	Apply simple tools for economic analysis
Teaching and mentoring	Train public health professionals; mentor public health professionals
Epidemiology of priority diseases and injuries	Evaluate and prioritize the importance of diseases or conditions of national public health concern

*Source: Traicoff et al. (*1*)

We found differences between this survey and our March–April 2020 survey (*11*) documenting FETPs’ contributions to COVID-19 preparedness and response. The response rate for this survey was lower than for the first (35% vs. 74%) (Table 2). Three (9%) programs responded to the second survey that had not responded to the initial survey: Mongolia FETP, Turkey FETP, and Somalia FETP. Among the 29 (91%) programs that responded to both surveys, more programs reported engagement of trainees and graduates than in the first survey. All programs responding to this second survey were well into COVID-19 response activities, having passed or approaching 1 year since COVID-19 introduction into their respective countries. This increase was noted across all WHO regions and pillars, underscoring the contributions of FETPs, its integration into national responses, and its adaptability through the engagement of FETPs in the new pillars. The themes that emerged in this survey were comparable to those identified in the first survey. The ongoing engagement of FETP trainees and graduates in COVID-19 response across all WHO regions and programs demonstrates FETPs’ value to ministries of health as a surge workforce to be leveraged in public health emergencies. Trainees and graduates were employed in their country’s response to the pandemic across the emergency response and preparedness pillars, and often in leadership roles.

The diverse, sustained, and increasing engagement of FETP trainees and graduates in COVID-19 responses around the world highlights FETPs’ far reach. WHO’s Joint External Evaluation (JEE) tool, developed to assess countries’ implementation of the IHR (2005), recognizes the importance of FETPs with a specific indicator (D.4.2 in JEE Tool version 1 and D.4.4 in version 2): FETP or other applied epidemiology training programs in place (*7*). Recent publications describe the discrepancy between JEE scores and outbreak response performance (*14*,*15*). One of Yemen’s highest JEE technical area score of 4 was in the workforce development indicator, stating that the country has “two levels of FETP or comparable applied epidemiology training programs in place in the country or in another country through an existing agreement.” However, the JEE assessment of IHR (2005) framework functions showed capacity to detect outbreaks but limited or no capacity to prevent or respond to them, reflecting that an FETP alone cannot yield an effective outbreak response. Our survey findings support that implementing FETPs could positively influence JEE results beyond the workforce development technical area, including the areas of emergency preparedness, emergency response operations, medical countermeasures, personnel deployment, risk communication, and points of entry. Engagement of FETP trainees and graduates in response operations and logistics, which are not FETP core competencies (Table 4), highlights the importance of regular assessments of the skills needed by the modern field epidemiologists or potential public health staffing gaps which FETPs may be filling (*1*).

We identified 4 limitations in the contribution of FETPs to COVID-19 preparedness and responses worldwide. First, the response rate to the second survey was about half that of the first (35% vs. 74%); responses from programs >20 years old were absent in most regions (EURO, PAHO, Southeast Asia Regional Office, and Western Pacific Regional Office). In the midst of the global pandemic, in the first quarters of 2021 when we conducted this follow-up survey, there were several factors that may account for the reduced response rate: program staff may have had limited time to respond to detailed surveys or to track graduates, and the expanded information requested made the second survey more time-intensive to complete. Second, FETP trainees and graduates bring diverse skillsets to the training, which limits our ability to attribute their contributions solely to their participation, particularly with regard to response pillar activities that do not align with FETP core competencies. Trainee and graduate engagement in pillars that did not require field epidemiologic competencies may be a function of either skills trainees had before enrolling in an FETP, skills they acquired elsewhere, seniority associated with career progression since FETP graduation, or a combination of those factors. Third, reporting bias is inherent to this documentation approach because of respondents’ motivation to inflate engagement of programs and their graduates. Quantifying the level of support needed by the trainees and graduates to participate effectively in response activities was beyond the scope of this effort. Finally, English was not the dominant language of some respondents. Misinterpretation of questions, inaccurate translations, and loss of nuance were possible. Nonetheless, the consistency of findings about engagement across the 2 surveys, in all WHO regions and response pillars, supports the importance of FETPs in countries preparing for and responding to public health threats.

This second documentation of FETPs’ contributions to responses to the COVID-19 pandemic highlights 3 needs in field epidemiology training. Systematic chronicling of how trainees, graduates, and program staff work to detect, respond, and prevent public health threats would help to build the body of evidence that field epidemiology training is valuable, and merits continued investment. Periodic tier-by-tier assessments could ensure that the skills developed through this training are the skills required by most field epidemiologists. Finally, regular updating of each tier of the FETP curriculum would assure that new skills required for field epidemiologists can be developed through FETPs. 

Future assessments of FETPs could include eliciting feedback from public health institutions on the quality of the contributions to the COVID-19 response of trainees, graduates, and staff. FETP evaluators can also engage with human-resource offices to ensure alignment of competencies with job requirements, pay scale, and a career path for epidemiologists. In addition, assessments can elicit self-reported information from FETP graduates about progression in their career attributable to training in field epidemiology.
